# The plasma proteome reveals markers of recent and repeated stress in free-ranging seals

**DOI:** 10.1093/conphys/coae075

**Published:** 2024-11-04

**Authors:** Jessica G Avalos, Cory D Champagne, Dan E Crocker, Jane I Khudyakov

**Affiliations:** Department of Biological Sciences, University of the Pacific, 3601 Pacific Ave., Stockton, CA 95211, USA; National Marine Mammal Foundation, 2240 Shelter Island Dr., Ste. 200, San Diego, CA 92106, USA; Biology Department, Sonoma State University, 1801 East Cotati Ave., Rohnert Park, CA 94928, USA; Department of Biological Sciences, University of the Pacific, 3601 Pacific Ave., Stockton, CA 95211, USA

**Keywords:** LC–MS/MS, plasma, proteome, seal, stress

## Abstract

Animals in nature potentially experience multiple stressors, and those of anthropogenic origin are likely to be repeated or chronic. However, stress hormone levels are highly context-dependent and are not consistent predictors of chronic stress in wildlife. Profiling the downstream consequences of repeated stress responses, such as changes in metabolism or gene expression, may be more informative for predicting their individual-level health consequences and population-level impacts, which are key objectives for wildlife conservation. We previously found that in free-ranging juvenile elephant seals, the blubber transcriptome and proteome, but not cortisol levels, could distinguish between responses to single versus repeated stress axis stimulation. However, the blubber proteome response to stress was limited and mainly involved extra-cellular matrix proteins. In this study, we examined the plasma proteome response of four of the same animals to the repeated stress experiment, since multiple organs secrete proteins into the circulation, providing a readout of their activity and integration. We isolated plasma proteins, identified and quantified them using liquid chromatography and tandem mass spectrometry (LC–MS/MS) and compared their abundance between sampling times. We identified >200 proteins in plasma, of which 42 were altered in abundance, revealing complex protein dynamics in response to repeated stress challenges. These changes were delayed but sustained, suggesting that the plasma proteome may reflect longer term integration of multi-organ responses to recent, rather than immediate, challenges. Differentially abundant proteins included components of the osmoregulatory system, acute phase and complement proteins, organokines, apolipoproteins and hormone transport proteins, which coordinate physiological processes with significant implications for marine mammal health and may explain several aspects of marine mammal stress physiology, such as insulin resistance and high aldosterone levels. We identified several potentially novel biomarkers, such as AGT, HPX, TTR and APOA4, that may be useful for detecting recent and repeated stress exposure in marine mammals.

## Introduction

The physiological response to acute stress is conserved and well defined in vertebrates and is mediated in part by the hypothalamic–pituitary–adrenal (HPA; or inter-renal, HPI in fish) axis (Romero and Gormally, 2019). In mammals, stress triggers secretion of corticotropin-releasing hormone by the hypothalamus, which stimulates the anterior pituitary gland to release adrenocorticotropic hormone (ACTH), which, in turn, induces synthesis of glucocorticoids (GCs; e.g. cortisol, corticosterone) by the adrenal cortex. GCs are transported by corticosteroid-binding globulin (CBG) in the circulation, and only free hormone that dissociates from CBG can enter cells, bind to intracellular glucocorticoid receptors (GR) in target tissues (e.g. adipose tissue, liver, muscle, immune cells) and alter expression of target genes (Sapolsky *et al.*, 2000; Breuner *et al.*, 2013). This leads to changes in protein abundance that mediate the physiological response to acute stress (e.g. release of energy substrates into the circulation via lipolysis, gluconeogenesis and proteolysis and suppression of immune system activity and reproductive function). These alterations are adaptive, enabling animals to survive immediate challenges (Sapolsky *et al.*, 2000).

However, animals in nature potentially experience multiple, simultaneous and/or sequential stressors that may have interactive effects (Côté *et al.*, 2016). Many anthropogenic stressors, such as sound disturbance, are increasing in prevalence and intensity and are more likely to be persistent (Kok *et al.*, 2023). Therefore, understanding wildlife responses to multiple and chronic stressors and predicting their individual- and population-level impacts have emerged as key priorities in conservation physiology (Pirotta *et al.*, 2018; Tyack *et al.*, 2022). Chronic stress has the potential to narrow an animal’s reactive scope, impacting its ability to maintain homeostasis, respond to further stressors, and complete key life history stages (Romero *et al.*, 2009). For example, chronic stress in marine mammals and other wildlife has been shown to impair immune and reproductive functions (DuRant *et al.*, 2016; Nabi *et al.*, 2018; Acevedo-Whitehouse, 2019), attenuate acute responses to additional stressors (Rich and Romero, 2005; Hanauer *et al.*, 2023) and alter the numbers of corticosteroid- and catecholamine-producing cells in the adrenal gland (Clark *et al.*, 2006). Unfortunately, GC levels, which are the most commonly used metric to detect stress in wildlife, are highly context-dependent and may be unreliable predictors of chronic stress, especially because chronically stressed animals may have attenuated GCs (Dickens and Romero, 2013; Dantzer *et al.*, 2014; Tablado and Jenni, 2017). The endocrinology of stress responses may even vary between closely related species; e.g. aldosterone and reverse triiodothyronine (rT3), which are not considered stress hormones *per se* in terrestrial mammals, are elevated during stress in marine mammals (Atkinson *et al.*, 2015; Champagne *et al.*, 2018; McCormley *et al.*, 2018). Therefore, identification of additional markers that provide taxon-specific information on the downstream consequences of physiological stress responses is critical for wildlife conservation (Gormally and Romero, 2020; Tyack *et al.*, 2022).

We recently described an experiment that simulated repeated stress in marine mammals using ACTH administration in juvenile northern elephant seals (NES; *Mirounga angustirostris*) (McCormley *et al.*, 2018). We targeted this life history stage as the animals are not moulting, reproducing or fasting extensively during this time and do not demonstrate associated variation in baseline corticosteroid and thyroid hormone levels (Kelso *et al.*, 2012; Jelincic *et al.*, 2017). Cortisol responses to each of four ACTH challenges, administered ~24 h apart, were highly variable between individuals and did not significantly differ between the first and fourth days. In contrast, aldosterone and total T3 levels, as well as the transcriptome and proteome of blubber tissue, were significantly altered by repeated ACTH challenges, providing additional markers (e.g. hormones other than GCs, genes associated with lipolysis and adipogenesis) for distinguishing responses to single versus multiple stressors (McCormley *et al.*, 2018; Deyarmin *et al.*, 2019; Deyarmin *et al.*, 2020). However, the blubber proteome response to ACTH was limited to few proteins that were primarily associated with connective tissue function, providing incomplete insights into the functional consequences of repeated stress responses (Deyarmin *et al.*, 2020).

In this study, we examined the plasma proteome response of the same animals to repeated ACTH administration. Virtually all vascularized organs, including those that cannot be sampled from free-ranging marine mammals (e.g. liver, heart, kidney), secrete proteins into the blood plasma, which thus reflects their secretory activity (Malmström *et al.*, 2016; Oh *et al.*, 2023; Tannouri and Simmons, 2023). Our previous work in NES showed that the plasma proteome was more responsive to prolonged physiological challenges than blubber or muscle (Khudyakov *et al.*, 2022), suggesting that this matrix is a rich source of biomarkers. We hypothesized that the plasma proteome would reflect integration of tissue responses to ACTH administration and provide valuable information about the indicators and impacts of recent and repeated stress in marine mammals.

## Materials and Methods

### Ethical declarations

All animal handling procedures were conducted under National Marine Fisheries Service permit 19 108 and approved by Sonoma State University and University of the Pacific Institutional Animal Care and Use Committees and Department of the Navy Bureau of Medicine and Surgery.

### Sample collection

We conducted a repeated ACTH administration experiment in juvenile (~0.8-year-old) NES in August–October 2016 at Año Nuevo State Park (San Mateo County, CA, USA) as described in McCormley *et al.*, 2018. The juvenile autumn haul-out period is not confounded by energetically demanding activities such as breeding or moulting or the changes in baseline corticosteroid or thyroid hormones that accompany these life history stages (Kelso *et al.*, 2012; Jelincic *et al.*, 2017). Study animals were selected based on apparent body condition indicating recent arrival at the rookery. For the current study, we used plasma samples from four animals (Seals 2 and 4, male; Seals 6 and 7, female) for which the blubber transcriptome and proteome responses to ACTH have been described (Deyarmin *et al.*, 2019; Deyarmin *et al.*, 2020).

**Figure 1:**
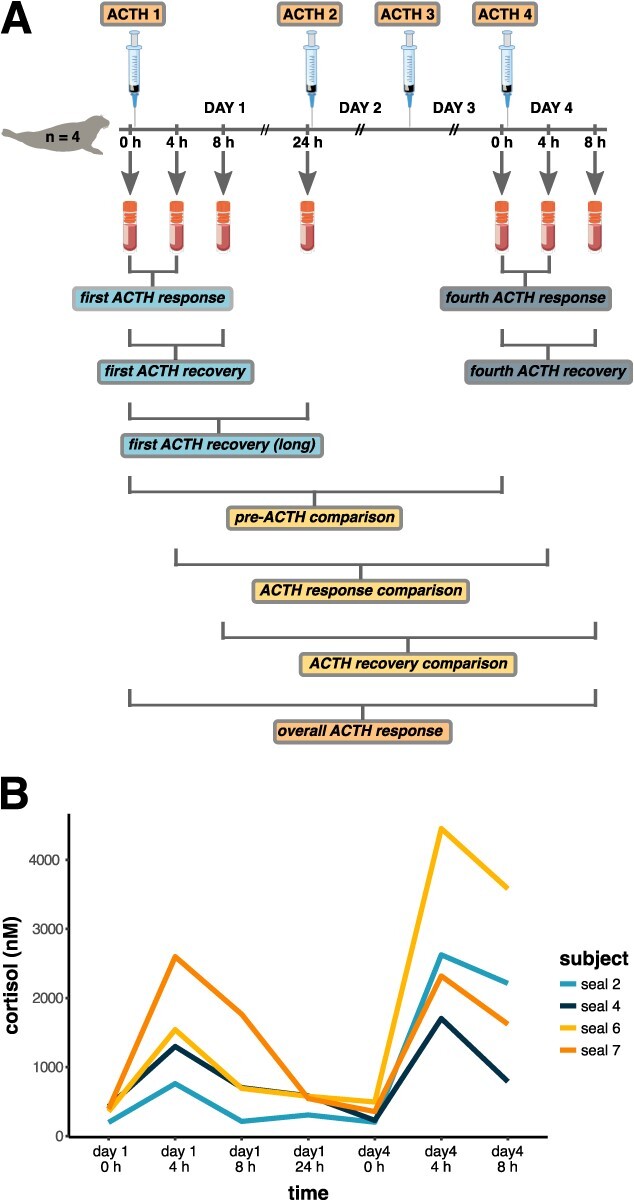
**A)** Overview of experimental design. ACTH was administered once daily for four days, ~24 h apart, to four juvenile northern elephant seals (NES). Blood samples were collected at the indicated time-points and plasma proteomes were compared as shown to examine responses to ACTH. Figure was modified from Deyarmin *et al.*, 2019. **B)** Serum cortisol levels measured in the four study animals (from McCormley *et al.*, 2018).

Seals were chemically immobilized using an intramuscular injection of 1 mg/kg tiletamine–zolazepam HCl, and sedation was maintained with intravenous doses of ketamine and diazepam. Previous work has shown that this anaesthesia procedure suppresses catecholamine and corticosteroid responses to research handling in NES (Champagne *et al.*, 2012). After the collection of baseline blood and blubber samples, 20 units of a synthetic ACTH preparation (mean mass-specific dose: 0.17 ± 0.02 U/kg) were administered via intramuscular injection into the posterior flank of each animal, once daily for four consecutive days (~24 h apart), to simulate repeated stress responses (McCormley *et al.*, 2018). Serial blood samples were collected pre-ACTH (0 h) and post-ACTH (4 and 8 h after ACTH administration) on Days 1 and 4, and pre-ACTH only on Day 2 (~22 h after the first ACTH administration) to assess the effects of repeated HPA axis stimulation on blood plasma proteomes (Fig. 1). Blubber samples were collected pre-ACTH and 4 h post-ACTH on Days 1 and 4 (four total samples), as previously described (Deyarmin *et al.*, 2019). Blood samples were obtained from the extradural vein using an 18 G, 3.25-in spinal needle within 18.0 ± 5.5 min of initial sedation. Samples were drawn directly into chilled EDTA-treated vacutainer tubes and were stored on ice in a field cooler for 1–3 h until processing. Plasma was isolated by centrifugation at 3000 × g for 15 min, kept frozen on dry ice until return to the laboratory and stored at −80°C until further analysis.

### Sample preparation for liquid chromatography and tandem mass spectrometry

All chemicals and reagents were obtained from VWR and Fisher Scientific (USA), unless otherwise indicated. We first compared four plasma protein sample preparation methods to determine denaturation and alkylation conditions that produced the largest number of identified peptides and proteins. We then used the top-performing method for all of the samples in this study, as outlined below. Method development details are provided in Supplementary File 1.

Six microliters of plasma were denatured for 1 h at 60°C in buffer containing 5% sodium deoxycholate (SDC) and 5 mM tris(2-carboxyethyl)phosphine (TCEP) in 50 mM ammonium bicarbonate (AmBiC; 200 μl final volume). Proteins were alkylated with 20 mM chloroacetamide (CAA) for 30 min in the dark at room temperature and quenched by addition of TCEP to a final concentration of 5 mM. Samples were diluted to reduce SDC concentration to 0.5% w/v and total protein concentration was estimated using the Pierce BCA Protein Assay Kit. Proteins were digested in solution using Thermo Scientific Trypsin Protease MS Grade at a 1:50 μg enzyme to protein ratio for 16 h at 37°C. After digestion, trifluoroacetic acid (TFA) was added to a final concentration of 1.0% v/v to inactivate trypsin and precipitate SDC; the latter was removed by centrifugation and extraction of the supernatant. Digested peptides were lyophilized, resuspended in 0.1% TFA and desalted using Pierce Peptide Desalting Spin Columns following the manufacturer’s protocol. Eluted peptides were lyophilized and resuspended in 0.1% formic acid in liquid chromatography–mass spectrometry (LC–MS)-grade water. Peptide concentrations were determined using Pierce Quantitative Colorimetric Peptide Assay (Thermo Fisher Scientific, USA) after 1:21 dilution in AmBiC to increase pH for the assay. Samples were analysed in duplicate (mean CV = 2.93%). Prism 9 (GraphPad, USA) was used to interpolate the standard curve (R^2^ = 0.999). Samples were diluted to a final concentration of 200 ng/μl for liquid chromatography and tandem mass spectrometry (LC–MS/MS).

### LC–MS/MS

Peptide samples were analysed by label-free quantification (LFQ) using high-performance liquid chromatography and tandem mass spectrometry (HPLC–MS/MS) as previously described (Khudyakov *et al.*, 2018). Three injections (technical replicates) were used for each sample. For each run, 5 μl of the sample (1 μg total) was loop injected onto a reversed-phase trap column (Acclaim PepMap 100 C18 LC column; 75 μm i.d. × 2 cm, 3 μm particle size, 100 A pore size) by a Dionex Ultimate 3000 autosampler. Peptides were eluted onto a reversed-phase analytical column set at 35°C for HPLC (EASY-SprayTM C18 LC column; 75 μm i.d. × 15 cm, 100 A). Solvent A was water and B was acetonitrile (ACN), respectively (both with 0.1% formic acid). During each chromatographic run, which lasted 140 min, flow rates were held at 300 nl/min. Solvent B (ACN) was used as follows: 2% at 5 min, 2–22% at 75 min, 22–38% at 100 min, 38–95% at 105 min, 95% returning to 2% at 115 min and lastly maintained at 2% at 140 min.

MS analysis was performed using data-dependent acquisition (DDA) on the Thermo Fisher Orbitrap Fusion™ Tribrid™ mass spectrometer equipped with an EASY-Spray™ ion source and operated by Xcalibur 4.0 software. MS1 spectra were resolved by the orbitrap with a resolution of 120 000, scan range of 200–1400 m/z, RF lens of 60%, AGC target of 1.0e6 and max injection time of 50 ms. Precursor ions were isolated by quadrupole and fragmented using HCD with a collision energy of 28% ± 3%. MS2 product ions were resolved by the orbitrap (resolution: 30 000, AGC target: 5.0e5, first mass: 100 m/z, max injection time: 150 ms).

### Statistical analyses

MS/MS data analyses were conducted using the MaxQuant v1.6.14.0 computational platform (Tyanova *et al.*, 2016). Peptides and proteins were identified using the UniProt SwissProt Mammalia database containing 67 420 proteins (Taxonomy ID: 40674, downloaded on 5 October 2021) and quantified using the MaxLFQ algorithm (Cox *et al.*, 2014). Median abundance values from three technical replicates were used for MaxLFQ. Default settings were used for most parameters, with the exception of three maximum missed trypsin cleavages and three maximum modifications per peptide. Oxidation (M) and acetyl (Protein N-term) were selected as variable modifications and carbamidomethylation (C) was selected as a fixed modification. The ‘match between runs’ option was used to align replicates during the run using default alignment settings.

Differential protein abundance analyses were conducted using the limma v3.50.0 package (Ritchie *et al.*, 2015) in R v4.1.0 (R Development Core Team, 2021). Missing protein abundance values were imputed for 153 proteins that had no more than two missing values per sample group (i.e. per time-point) using the *nni* function in the pcaMethods v1.96.0 package (Välikangas *et al.*, 2017). Differential protein abundance analyses were conducted with blocking by sample processing batch and inclusion of a within-individual correlation term to account for repeated sampling (model design = ~ time + batch; block = subject; correlation = consensus.correlation), as suggested by the limma User’s Guide section 9.7 (Smyth *et al.*, 2005). *P*-values were adjusted for multiple hypothesis testing using Benjamini–Hochberg correction and were considered significant at adjusted *P* < 0.1. Heat maps were generated using the pheatmap v1.0.12 package with scaling by row and complete clustering of columns and rows by Euclidean distance using Ward’s minimum variance method (Ward D2) (Murtagh and Legendre, 2014). Abundances of multiple isoforms of the same proteins were averaged to investigate overall changes in protein abundance across the experimental study period (Fig. 3).

## Results

**Figure 2:**
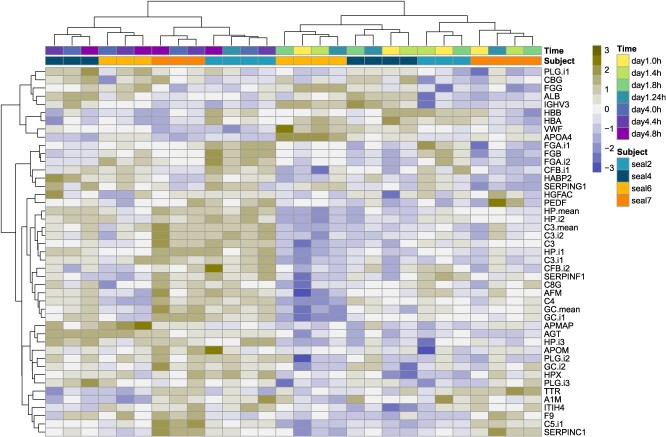
Heat map showing log2-transformed and scaled abundance of differentially abundant plasma proteins (rows) in juvenile northern elephant seals (NES, *n* = 4) sampled during a repeated ACTH administration experiment (columns). Rows and columns were hierarchically clustered by Euclidean distance using Ward’s D2 minimum variance method to emphasize proteins with similar abundance patterns. Protein abundance relative to all 28 samples is indicated with the colour scale shown in the legend.

We profiled the plasma proteomes of four juvenile NES sampled over a repeated ACTH administration experiment lasting four days and compared protein abundance between sampling points to determine responses to repeated HPA axis activation (Fig. 1). We identified 231 proteins with one or more unique peptides, of which 153 were used for differential abundance analyses (see Methods; Supplementary File 2). Global protein abundance showed high individual variability in responses following ACTH (Supplementary Fig. S1). Clustering analysis showed that protein abundance varied most significantly between Days 1 and 4, whereas within each day, it clustered more strongly by subject (Fig. 2). Consequently, we did not detect significant changes in protein abundance within 24 h of the first ACTH administration or within 8 h of the fourth ACTH administration (adj. *P* > 0.1) and only identified differentially abundant proteins (DAPs; *n* = 42 total unique proteins) in comparisons between days (Fig. 2).

### Differentially abundant proteins in response to repeated ACTH administration

We identified 8 DAPs in the pre-ACTH comparison, 15 DAPs in the ACTH response comparison, 20 DAPs in the ACTH recovery comparison and 34 DAPs in the overall comparison, i.e. between Day 1 pre-ACTH and Day 4 ACTH recovery (Table 1, Fig. 2, Supplementary File 3). Five DAPs were common to all comparisons: a component of the renin–angiotensin–aldosterone system (RAAS) (angiotensinogen, AGT), an acute phase protein (APP) (haptoglobin, HP) and complement proteins C3 and C5 increased, whilst apolipoprotein A-IV (APOA4) decreased in abundance on Day 4 relative to Day 1 (Fig. 2). The adipogenesis regulator APMAP (adipocyte plasma membrane-associated protein) increased on Day 4 relative to Day 1 in all comparisons except pre-ACTH. Several proteins with functions of interest to endocrine and metabolic physiology were altered in two of the four comparisons. These included vitamin D-binding protein (DBP, also known as ‘GC’; increased on Day 4 in pre-ACTH and overall response comparisons), apolipoprotein M (APOM; increased on Day 4 in ACTH recovery and overall response comparisons) and thyroid hormone transport protein transthyretin (TTR; decreased on Day 4 in ACTH response and ACTH recovery comparisons).

**Table 1:** Plasma proteins that were altered in abundance (DAP) in response to repeated ACTH administration in juvenile northern elephant seals (NES, *n* = 4). Pairwise comparisons were made as follows. Pre-ACTH comparison: Day 4 pre-ACTH versus Day 1 pre-ACTH; ACTH response comparison: Day 4 4 h post-ACTH versus Day 1 4 h post-ACTH; ACTH recovery comparison: Day 4 8 h post-ACTH versus Day 1 8 h post-ACTH; overall ACTH response: Day 4 8 h post-ACTH versus Day 1 pre-ACTH. Protein ID refers to the unique identifier used for each protein in this study. FC: fold change. Putative functions were obtained from the UniProt database. Complete results output for DAP lists can be found in Supplementary File 3.

**Protein ID**	**Protein name**	**FC**	**Putative function**
***Pre-ACTH comparison***			
SP108 SP15	haptoglobin (HP)	1.97 1.69	acute phase response
SP109	angiotensinogen (AGT)	1.75	RAAS component
SP88	complement C5 (C5)	1.38	complement system
SP61 SP23	complement C3 (C3)	1.33 1.23	complement system
SP207	vitamin D-binding protein (DBP)	1.30	vitamin D transport
SP1	apolipoprotein A4 (APOA4)	−2.25	lipid transport
***ACTH response comparison***			
SP227 SP108 SP15	haptoglobin (HP)	1.80 1.74 1.66	acute phase response
SP71	hemopexin (HPX)	1.43	acute phase response
SP118	inter-alpha-trypsin inhibitor heavy chain H4 (ITIH4)	1.51	acute phase response
SP70	adipocyte plasma membrane-associated protein (APMAP)	1.66	adipogenesis
SP109	angiotensinogen (AGT)	1.62	RAAS component
SP88	complement C5 (C5)	1.55	complement system
SP39 SP23	complement C3 (C3)	1.27 1.91	complement system
SP113	albumin (ALB)	1.60	fatty acid and ion transport
SP86 SP30	plasminogen (PLG)	1.39 1.30	blood coagulation
SP92	transthyretin (TTR)	−1.58	thyroid hormone and retinol transport
SP1	apolipoprotein A4 (APOA4)	−2.34	lipid transport
***ACTH recovery comparison***			
SP108 SP15 SP227	haptoglobin (HP)	2.17 1.59 1.55	acute phase response
SP70	adipocyte plasma membrane-associated protein (APMAP)	1.99	adipogenesis
SP218	apolipoprotein M (APOM)	1.97	lipid transport
SP109	angiotensinogen (AGT)	1.54	RAAS component
SP88	complement C5 (C5)	1.42	complement system
SP39 SP61 SP23	complement C3 (C3)	1.32 1.27 1.25	complement system
SP123	hyaluronan-binding protein 2 (HABP2)	1.41	blood coagulation
SP195	plasminogen (PLG)	1.41	blood coagulation
SP74	plasma protease C1 inhibitor (SERPING1)	1.31	blood coagulation

**Table 1:** Continued

**Protein ID**	**Protein name**	**FC**	**Putative function**
SP57	fibrinogen beta chain (FGB)	1.27	blood coagulation
SP40	fibrinogen alpha chain (FGA)	1.25	blood coagulation
SP44	von Willebrand factor (VWF)	−1.45	blood coagulation
SP55	immunoglobulin heavy variable 3–23 (IGHV3–23)	−1.26	immune function
SP92	transthyretin (TTR)	−1.37	thyroid hormone and retinol transport
SP228	alpha-1-macroglobulin (A1M)	−1.53	acute phase response
SP1	apolipoprotein A4 (APOA4)	−2.28	lipid transport
***Overall ACTH response***			
SP109	angiotensinogen (AGT)	1.84	RAAS component
SP70	adipocyte plasma membrane-associated protein (APMAP)	1.84	adipogenesis
SP15 SP108 SP227	haptoglobin (HP)	1.75 1.74 1.49	acute phase response
SP218	apolipoprotein M (APOM)	1.69	lipid transport
SP88	complement C5 (C5)	1.46	complement system
SP61 SP23 SP39	complement C3 (C3)	1.46 1.31 1.31	complement system
SP145	complement component C8 gamma chain (C8G)	1.42	complement system
SP35 SP58	complement factor B (CFB)	1.37 1.36	complement system
SP12	complement 4A (C4A)	1.29	complement system
SP69	hepatocyte growth factor activator (HGFAC)	1.43	hepatokine signalling
SP240 SP67	pigment epithelium-derived factor (PEDF, or SERPINF1)	1.38 1.33	hepatokine signalling
SP9	afamin (AFM)	1.20	hepatokine signalling
SP160	corticosteroid-binding globulin (CBG, or SERPINA6)	1.32	corticosteroid transport
SP207 SP41	vitamin D-binding protein (DBP)	1.28 1.26	vitamin D transport
SP146	fibrinogen gamma chain (FGG)	1.81	blood coagulation
SP232 SP40	fibrinogen alpha chain (FGA)	1.39 1.25	blood coagulation
SP57	fibrinogen beta chain (FGB)	1.34	blood coagulation
SP195 SP86	plasminogen (PLG)	1.55 1.42	blood coagulation
SP28	coagulation factor IX (F9)	1.54	blood coagulation
SP123	hyaluronan-binding protein 2 (HABP2)	1.28	blood coagulation
SP203	antithrombin-III (SERPINC1)	1.22	blood coagulation
SP44	von Willebrand factor (VWF)	−1.35	blood coagulation
SP106	haemoglobin subunit beta (HBB)	−1.55	oxygen transport
SP61	haemoglobin subunit alpha (HBA)	−1.92	oxygen transport
SP1	apolipoprotein A4 (APOA4)	−2.16	lipid transport

We also identified proteins that were unique to each comparison. In the ACTH response comparison, albumin (ALB), heme scavenger hemopexin (HPX) and acute phase protein ITIH4 increased on Day 4 relative to Day 1. In the ACTH recovery comparison, the clotting protein SERPING1 increased, whilst acute phase protein alpha-1-macroglobulin (A1M) and immunoglobulin IGHV3 decreased on Day 4 relative to Day 1. Proteins identified only in the overall response comparison included CBG (also known as SERPINA6), Wnt carrier protein afamin (AFM), hepatocyte growth factor activator (HGFAC), pigment epithelium-derived factor (PEDF, also known as SERPINF1), three complement proteins (C4, C8G, CFB) and several clotting proteins (F9, FGG, SERPINC1), which all increased on Day 4 relative to Day 1. Proteins unique to this comparison that decreased on Day 4 compared to Day 1 were haemoglobin subunits A and B (HBA, HBB).

### Trends in protein abundance over time

**Figure 3:**
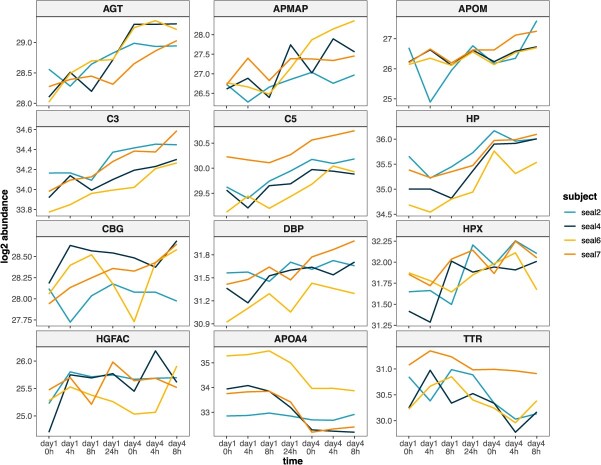
Log-transformed abundances of proteins of interest that were altered following four repeated ACTH challenges in four juvenile northern elephant seals (NES). Protein abundances are shown across all sampling points for each of the study subjects. Abundances were averaged for proteins with multiple isoforms (C5, DBP, HP). AGT: angiotensinogen; APMAP: adipocyte plasma membrane-associated protein; APOM: apolipoprotein M; C3: complement C3; C5: complement C5; HP: haptoglobin; CBG: corticosteroid-binding globulin; DBP: vitamin D-binding protein; HPX: hemopexin; HGFAC: hepatocyte growth factor activator; APOA4: apolipoprotein A4; TTR: transthyretin.

To examine the time-course of plasma protein abundance changes and degree of individual variability in responses following ACTH, we visualized trends in DAP abundance across all sampling points of the experiment, even if they were not considered significantly different in every comparison (Fig. 3). AGT, APOM, C3, C5, HPX and APOA4 showed the most consistent changes in abundance across study subjects following repeated ACTH administration (Fig. 3). Seal 2 appeared to be an outlier in its response to the experiment, showing either steep decreases in protein abundance within 4 h of the first ACTH administration (AGT, APMAP, APOM, CBG, TTR) or no change in abundance (APOA4), which contrasted with trends in the other subjects. We noted that several proteins showed rapid, transient changes in abundance following ACTH administration, especially on Day 1, increasing 4 h post-ACTH and returning towards baseline levels within 8 h, or *vice versa* (e.g. APMAP, APOM, C5, HP, HPX, HGFAC). However, all of the markers also showed a delayed, potentially secondary response following the first ACTH challenge, increasing (or decreasing, in the case of APOA4 and TTR) between 8 and 24 h of the first ACTH dose. Marker responses following the fourth ACTH administration were variable; several proteins that were altered following ACTH administration on Day 1 showed no change or a decrease in abundance between pre- and post-ACTH on Day 4 (AGT, C5, HP, APOA4).

## Discussion

This study describes the plasma proteome response to a repeated ACTH administration experiment in free-ranging juvenile NES, complementing published endocrine profiles and blubber transcriptome and proteome data from the same animals (McCormley *et al.*, 2018; Deyarmin *et al.*, 2019; Deyarmin *et al.*, 2020). We identified >200 proteins in plasma, of which 42 were altered in abundance between Days 1 and 4 of the experiment, revealing complex protein abundance dynamics and differences between responses following the first and subsequent ACTH challenges. Fifteen DAP identified in this study were also altered in tandem with baseline cortisol elevation during fasting in NES (Khudyakov *et al.*, 2022), including AGT, APOA4, APOM, C3, C5 and HPX, suggesting that that they may be potential markers of both baseline and stress-induced HPA axis activation in marine mammals.

The limited sample size of this study and high variability in individual responses to ACTH reduced the power to detect small changes in protein abundance within 4–8 h of each administration, despite concurrent changes identified within 4 h in the blubber transcriptome and proteome of the same animals (Deyarmin *et al.*, 2019; Deyarmin *et al.*, 2020). This variability did not appear to be driven by sex, although our sample size did not enable comparisons between proteome profiles of males and females. A larger sample size will be necessary to confirm trends in plasma protein abundance changes within 4 h of ACTH administration shown in Fig. 3 (e.g. potential increases in APOM, CBG, HGFAC and TTR abundance). Nevertheless, we identified significant and sustained changes in plasma protein abundance between the first and fourth day of the experiment, including between samples that were not distinguishable by cortisol levels alone (McCormley *et al.*, 2018). This suggests that the plasma proteins identified in this study may be promising markers of recent and repeated stress, which we broadly group into six functional categories with implications for marine mammal physiology and health.

### Renin–angiotensin–aldosterone system

AGT, which is primarily known for its role in regulation of blood pressure and osmolarity as the inactive precursor of the RAAS (Skov *et al.*, 2014), significantly increased between the first and fourth ACTH administration. In humans and rodents, the liver is the main source of circulating AGT, followed by adipose tissue; a number of other tissues (e.g. adrenal cortex, heart, kidney) also produce AGT locally. AGT abundance is regulated transcriptionally, and transcription of the *AGT* gene is activated by multiple hormones, including GCs, aldosterone and oestrogen (Demura *et al.*, 2015). *AGT* mRNA abundance also increased in response to ACTH in blubber of the same animals (Deyarmin *et al.*, 2019), suggesting that blubber may be a source of elevated AGT protein in plasma. An increase in abundance of AGT under conditions that activate RAAS may cause an increase in angiotensin II, which is a potent vasoconstrictor and inducer of aldosterone synthesis by the adrenal cortex. This, along with ACTH, which also stimulates aldosterone synthesis, may contribute to the dramatic increase in aldosterone observed in response to ACTH administration in NES (McCormley *et al.*, 2018). AGT protein abundance and aldosterone levels also increase (to a lesser extent) during fasting in NES, which suggests that activation of RAAS may be a common feature of increases in baseline- and stress-induced HPA axis activity in marine mammals (Ortiz *et al.*, 2000; Ortiz *et al.*, 2006; Champagne *et al.*, 2018; DeRango *et al.*, 2019; Khudyakov *et al.*, 2022). Whilst RAAS activation may serve as an adaptive mechanism to conserve water during fasting or acute stress, chronic stress may have adverse consequences for diving mammals, e.g. by impacting cardiovascular adjustments to apnea and increasing reactive oxygen species (ROS) production via RAAS hyperactivation (Masi *et al.*, 2019).

### Organokines

Repeated ACTH administration increased plasma abundance of four organokines (APMAP, PEDF, AFM, HGFAC) or signalling proteins produced by organs such as liver, adipose and muscle. APMAP is produced by liver, adipose tissue and endocrine organs and is involved in adipogenesis, a process that is stimulated by GCs (Bogner-Strauss *et al.*, 2010). The increased abundance of plasma APMAP was consistent with blubber transcriptome and proteome data from the same animals, which showed that multiple genes and proteins associated with adipogenesis were upregulated in response to repeated ACTH administration (Deyarmin *et al.*, 2019; Deyarmin *et al.*, 2020). In NES, an increase in the number of mature, functional adipocytes that are capable of responding to hormone signals may increase the capacity for lipid mobilization by lipolysis, which increases in response to elevated stress hormones and fasting in seals (Crocker *et al.*, 2014; Debier *et al.*, 2020). APMAP also functions as a paraoxonase-type anti-oxidant, in part by reducing membrane lipid oxidation and ceramide levels (Paul *et al.*, 2024). Recent work showed that the function of a key mammalian paraoxonase family member, PON1, has been lost in marine mammals, including NES (Meyer *et al.*, 2018). Our study suggests the intriguing possibility that the anti-oxidant function of PON1 is compensated by APMAP in marine mammals.

PEDF is a signalling molecule produced by adipose tissue, liver and muscle that promotes insulin resistance and stimulates lipolysis (Borg *et al.*, 2011; Lőrincz *et al.*, 2023). AFM is a hepatokine that is positively correlated with insulin resistance in humans (Lőrincz *et al.*, 2023). HGFAC is a protease produced by hepatocytes that activates hepatocyte growth factor (HGF), a multi-functional hormone that plays a key role in liver repair and regeneration as well as lipid and glucose homeostasis (Sargsyan *et al.*, 2023). An increase in the abundance of these organokines in response to ACTH suggests potential mechanisms by which cortisol induces its hallmark metabolic effects—stimulation of lipolysis and insulin resistance.

### Acute phase proteins and haemoglobin

Three proteins associated with the acute phase response (HP, HPX, A1M), a systemic response to infection, inflammation or injury, increased following repeated ACTH administration, with HP showing highly consistent elevation between Days 1 and 4. It is possible that the increase in APP abundance observed in this study was caused in part by repeated blood and biopsy sampling, rather than ACTH alone, as the changes in HP abundance followed the time-course of the acute phase response to tissue injury seen in other species (Kataranovski *et al.*, 1999). However, plasma HP was also elevated in adult female NES treated with ACTH compared to controls treated with saline (correcting for the effects of research handling); both groups were blood-sampled only once and not biopsied (J. Khudyakov, unpublished data). In addition, studies in other systems showed that APPs can be induced by GCs (Speelman *et al.*, 2022). This suggests that plasma levels of APPs such as HP may serve as alternative or additional metrics of stress in marine mammals. HP and HPX are involved in clearance of free haemoglobin and heme, respectively, which are released during erythrocyte turnover and hemolysis (Schaer *et al.*, 2014). A1M also binds heme, reduces oxidized biomolecules and scavenges free radicals (Bergwik *et al.*, 2021). Therefore, these proteins function as anti-oxidants by preventing heme toxicity.

The increase in HP, HPX and A1M abundance was coupled with a decrease in abundance of two haemoglobin (Hb) subunits, HBA and HBB, potentially as a consequence of increased Hb clearance by APPs (Vallelian *et al.*, 2010). Alternatively, the decrease in Hb may be the consequence of thyroid axis suppression, which was observed in these animals (McCormley *et al.*, 2018), as T3 stimulates both Hb synthesis and erythropoiesis (Park *et al.*, 2017). An increase in plasma HPX was also coupled with a decrease in HBA over fasting in NES, which is accompanied by baseline GC elevation (Khudyakov *et al.*, 2022). During prolonged fasting, blood volume stays isometric with body size, so an increase in Hb turnover may be a fasting adaptation for maintaining constant blood volume whilst undergoing reductions in mass (Somo *et al.*, 2015). Further functional work will be necessary to test whether acute or repeated stress responses alter erythropoiesis or Hb turnover in diving mammals. Nevertheless, increased levels of APPs may serve as promising indicators of repeated or sustained stress in marine mammals, and their measurement by LC–MS/MS presents a potential solution to some of the challenges of other APP detection methods (Gelain and Bonsembiante, 2019). However, since we used relative, rather than absolute, quantification of proteins in this study, optimization of different MS acquisition methods (e.g. selected reaction monitoring) will be necessary to develop MS-based quantitative APP assays. In addition, further studies should carefully examine the impact of repeated blood and biopsy sampling on these and other inflammatory markers (such as complement proteins) in marine mammals.

### Complement proteins

Five members of the complement system (C3, C4, C5, C8G, CFB), which is comprised of a cascade of liver-derived plasma proteins (as well as a system of intra-cellular proteins) that is rapidly activated by pathogens, apoptotic cells, tissue damage or foreign materials, increased following repeated ACTH administration. As for APPs, we cannot exclude the possibility that the increase in complement protein abundance was influenced in part by repeated animal sampling. Upon activation, enzymatic cleavage of C3 and C5 produces biologically active peptides that mark pathogens for immune phagocytosis, directly damage their cell membranes (in concert with C8) and stimulate an inflammatory response by other immune components. C4 is also cleaved during pathway activation, and its products bind to pathogens and participate in an enzyme complex (along with CFB) that activates C3, amplifying the complement cascade (Merle *et al.*, 2015). Whilst the anti-inflammatory effects of cortisol are well known and include the inhibition of complement activation, recent studies showed that the highly conserved complement pathway is also activated during both acute and chronic stress in fish (Samaras *et al.*, 2016; Demers and Bayne, 2020; Raposo de Magalhães *et al.*, 2020). We also recently showed that several genes encoding complement proteins (e.g. C1Q, C9) were upregulated in NES blubber tissue in response to 48 h of cortisol treatment *ex vivo*, suggesting that GCs may enhance complement responses in marine mammals as well (Kashiwabara *et al.*, 2023). An increase in complement protein abundance in response to stress may enhance an animal’s immunocompetence, but excessive complement pathway activity has been associated with tissue damage and hemolysis in humans (Merle *et al.*, 2015); whether this is likely in a wild animal is unknown. We recommend that future studies use targeted assays to examine whether cortisol stimulates complement protein activity, as we were unable to distinguish between complement protein cleavage products by proteomics (e.g. proteins identified as C3 included peptides annotated as both C3A and C3B). Such studies should also include controls to determine whether sample collection protocols impact complement levels.

### Hormone transport proteins

Abundance of cortisol transporter CBG and vitamin D transporter DBP increased, whilst that of TTR, which transports thyroxine and retinol-binding protein, decreased following repeated ACTH administration. CBG (measured by ELISA) also increased in parallel with cortisol across fasting in adult male NES (D. Crocker, unpublished observation). Since cortisol that is bound to CBG is unable to enter cells, an increase in CBG concentration may indicate buffering of the effects of prolonged baseline cortisol elevation (Breuner *et al.*, 2013). However, the functional significance of an increase in plasma CBG in response to ACTH will require determination of CBG–cortisol binding affinity in this species in order to calculate the concentration of free, and therefore biologically relevant, cortisol in the circulation (Delehanty *et al.*, 2015).

The expression of *DBP* in mammals is regulated in part by GCs. In humans, DBP was shown to be necessary for glucagon secretion during metabolic stress (Bikle and Schwartz, 2019; Viloria *et al.*, 2022). Since blood glucose levels increase in response to ACTH administration in NES (Champagne *et al.*, 2015; Khudyakov *et al.*, 2015), as in other mammals, the increase in DBP protein abundance observed in this study suggests a potential mechanism by which cortisol may synergize with glucagon to increase glucose levels during stress responses.

The abundance of thyroxine-binding protein TTR decreased following ACTH administration and mirrored the decrease in total T3 levels reported in the same animals (McCormley *et al.*, 2018). The effect of the decrease in TTR abundance on free thyroid hormone levels is unclear, since only <30% of thyroxine (the precursor to T3) is bound by TTR; the majority is transported by thyroxine-binding globulin (TBG) (Richardson, 2007). However, we did not detect TBG in the NES plasma proteome. It is possible that a decrease in TTR may increase the concentration of free T4, thereby counteracting the decrease in total T3 levels observed in our study. However, rT3, the inactive form of thyroid hormone, also increases in parallel with cortisol in NES (McCormley *et al.*, 2018; Peterson *et al.*, 2023). This suggests that HPA axis activation may cause an overall decrease in the pool of biologically active T3. *TTR* expression in human liver is under negative acute phase regulation, meaning that it is inhibited in response to stimuli that normally activate APPs (Richardson, 2007). Accordingly, the decrease in TTR was accompanied by an increase in APP abundance in our study. TTR may have other functions with significance for marine mammal stress physiology, as it also transports RBP4, an adipokine associated with insulin resistance (Yang *et al.*, 2005). A decrease in TTR may increase free RBP4 levels, promoting insulin resistance during stress responses. Whilst we identified RBP4 in the NES plasma proteome, its abundance did not change significantly following ACTH administration, and further research will be necessary to determine the functional consequences of changes in TTR levels.

### Apolipoproteins

Repeated ACTH administration altered the abundance of two apolipoproteins: APOM, which increased, and APOA4, which decreased between Days 1 and 4. APOM is produced by the liver, kidney and adipose tissue and is a component of mature high-density lipoproteins. In mice, APOM inhibits lipid uptake by adipocytes and promotes insulin resistance (Hajny *et al.*, 2021). In contrast, APOA4 is a constituent of triglyceride-rich lipoproteins; it mediates intestinal absorption of dietary lipids and cellular uptake of fatty acids, stimulates insulin secretion and decreases hepatic gluconeogenesis (Qu *et al.*, 2019). Plasma APOM also increased and APOA4 decreased during fasting in NES (Khudyakov *et al.*, 2022), and these changes appear to be a consistent response to ACTH administration during other life history stages in seals (J. Khudyakov, unpublished observation). Therefore, apolipoproteins have potential as markers of food restriction in marine mammals during prolonged fasting or HPA axis responses to repeated disturbance.

## Conclusions

In summary, we identified delayed, but sustained changes in the plasma proteome in response to repeated ACTH administration in juvenile NES. High individual variability in animal responses to ACTH precluded identification of immediate, short-term changes in the plasma proteome, which may become apparent in a larger study. This suggests that unlike the transcriptional and proteomic responses of individual tissues (e.g. blubber) to ACTH, which were detectable within several hours of administration, the plasma proteome may be more suitable for examining longer-term integration of multi-organ responses to recent, rather than immediate, challenges (Gormally and Romero, 2020). The high temporal resolution provided by repeated sampling over 4 days in this study offered a unique opportunity to examine proteome dynamics and multi-phase responses to the experiment (i.e. immediate vs second- and third-order responses). We found that following repeated ACTH administration, the abundance of components of the RAAS, acute phase and complement proteins, hepatokines and adipokines, hormone transport proteins and apolipoproteins were altered in plasma. These proteins, which included AGT, HPX, TTR and APOA4, coordinate multiple physiological processes, including lipid metabolism, cardiovascular and renal function, innate immune response, haemoglobin recycling, cell maturation and turnover and endocrine signalling. Therefore, their abundance, when assessed in the context of life history, may provide information about an animal’s allostatic load.

High variance in endocrine and proteome responses to ACTH, even amongst conspecifics of similar age and apparent body condition, highlights the need to take individual stress reactivity into consideration (Schoenle *et al.*, 2018). Furthermore, stress responses must be evaluated in the context of life history, which influences HPA axis reactivity and may have additive effects on allostatic load (Crespi *et al.*, 2013). The magnitude and duration of GC responses to ACTH varies by life history stage in NES, which fast extensively during post-natal development, breeding and moulting (Ensminger *et al.*, 2014; Champagne *et al.*, 2015; Ensminger *et al.*, 2021). In this study, we attempted to decouple responses to exogenous HPA axis activation from physiological adjustments to life history challenges by targeting NES during a brief haul-out period that does not involve extensive fasting and high energy expenditure. However, future studies should examine both baseline changes in the plasma proteome across life history, which has only been done in moulting adult female NES to date (Khudyakov *et al.*, 2022), and their responses to HPA axis stimulation during different life history stages to properly contextualize protein markers and assess their diagnostic utility. In summary, we suggest that the plasma proteome has the potential to provide information about recent exposure to multiple and/or repeated stressors in marine mammals and their potential effects on health, reproduction and survival, filling a key mechanistic gap in assessments of impacts of anthropogenic activities on marine ecosystems (Pirotta *et al.*, 2018; Tyack *et al.*, 2022).

## Supplementary Material

Web_Material_coae075

## Data Availability

Raw MS/MS data is available as dataset PXD054543 in the ProteomeXchange MassIVE database: ftp://massive.ucsd.edu/v08/MSV000095508/
