# Supplementary material for: The plasma proteome reveals markers of recent and repeated stress in free-ranging seals
Source: Conserv Physiol. 2024 Nov 4;12(1):coae075. doi: 10.1093/conphys/coae075 (PMC11533252; doi:10.1093/conphys/coae075)
Supplement: Web_Material_coae075 [file web_material_coae075.zip › SupplementaryFile1_R1.pdf]

The plasma proteome reveals markers of recent and repeated stress in free-ranging seals
Avalos J.G., Champagne C.D., Crocker D.E., Khudyakov J.I.

Supplementary Information

Supplementary Methods

We previously used 8 M urea and 10 mM dithiothreitol (DTT) for solubilization, denaturation, and reduction of protein pellets remaining after RNA extraction from seal tissues and iodoacetamide (IAA) for protein alkylation (Deyarmin *et al.*, 2020, Khudyakov *et al.*, 2018). However, recent studies have shown that urea, DTT, and IAA lead to protein modifications during sample preparation that may reduce peptide and protein identification rates (Müller *et al.*, 2017, Proc *et al.*, 2010, Wojdyla *et al.*, 2015). Alternatively, denaturation with sodium deoxycholate (SDC), reduction with tris(2-carboxyethyl)phosphine (TCEP), and alkylation with chloroacetamide (CAA) may increase protein and peptide identification rates (León *et al.*, 2013, Scheerlinck *et al.*, 2015). We used baseline plasma samples collected from juvenile seals (n = 2) during a different study for method development (Pujade Busqueta *et al.*, 2020). Denaturing buffer was added to 6 uL (~300 ug protein) of each plasma sample to a final volume of 200 uL. Denaturation, reduction, and alkylation were conducted as following:

Urea/DTT/IAA protocol used a denaturing buffer composed of 8 M urea and 10 mM dithiothreitol (DTT) in 50 mM ammonium bicarbonate (AmBiC). Samples were incubated at 30°C for 1 hour. Proteins were alkylated with 20 mM iodoacetamide (IAA) for 30 minutes in the dark at room temperature. Alkylation was quenched by the addition of DTT to a final concentration of 10 mM.

Urea/TCEP/CAA protocol used a denaturing buffer solution composed of 8 M urea and 5 mM TCEP in 50 mM AmBiC. Samples were incubated at 30°C for 1 hour. Proteins were alkylated with 20 mM chloroacetamide (CAA) for 30 minutes in the dark at room temperature and quenched by addition of TCEP to a final concentration of with 5 mM.

SDC/DTT/IAA protocol used a denaturing buffer composed of 5% w/v SDC and 10 mM DTT in 50 mM AmBiC. Samples were incubated at 60°C for 1 hour. Proteins were alkylated with 20 mM IAA for 30 minutes in the dark at room temperature and quenched by the addition of DTT to a final concentration of 10 mM.

SDC/TCEP/CAA protocol used a denaturing buffer solution composed of 5% w/v SDC and 5 mM TCEP in 50 mM AmBiC. Samples were incubated at 60°C for 1 hour. Proteins were alkylated with 20 mM CAA for 30 minutes in the dark at room temperature and quenched by addition of TCEP to a final concentration of with 5 mM.

The SDC/TCEP/CAA protocol produced marginally higher numbers of identified peptides and identified proteins with non-zero abundance compared to the other protocols, although the differences were not significant (peptides: p = 0.09; proteins: p = 0.22; data not shown). We selected this protocol for further sample processing due to its simplicity and lower risk of potential off-target modifications, as has been shown in other studies (León *et al.*, 2013, Scheerlinck *et al.*, 2015). Samples were randomized and processed in two batches of n = 14 each. See main text for information about the rest of the sample preparation protocol.

Supplementary Figures

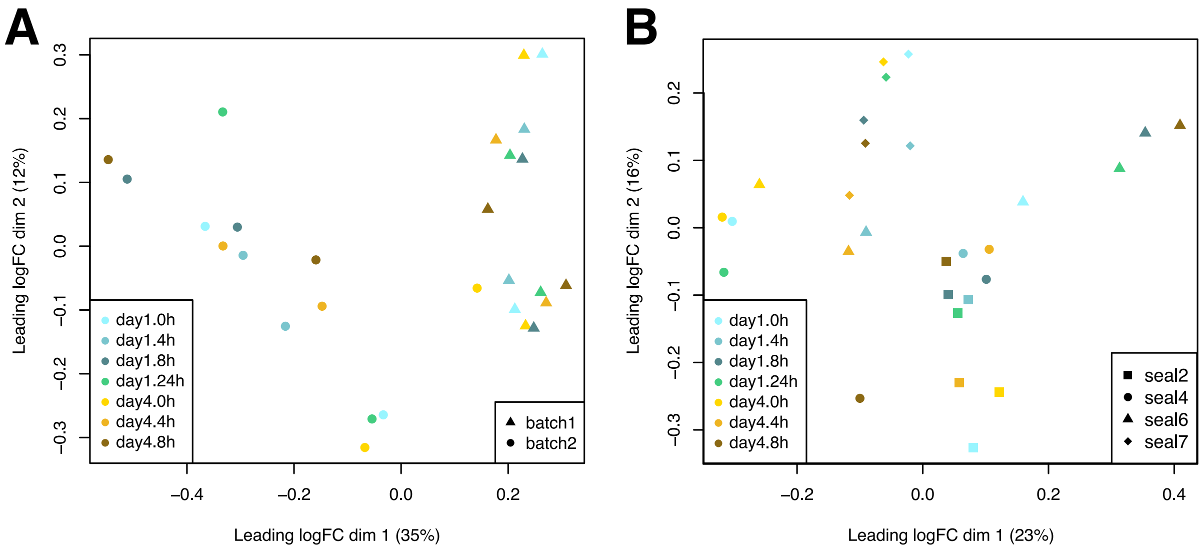

Supplementary Figure 1. Multi-dimensional scaling (MDS) plots showing global plasma protein abundance in four juvenile northern elephant seals (*Mirounga angustirostris*) sampled following a repeated ACTH administration experiment. Points are color-coded by sampling time. **A)** Point shape denotes batch in which samples were processed for LC-MS/MS analyses. **B)** MDS plot of data corrected for batch effect, with point shapes denoting study individuals.

Description of Supplementary Data

Supplementary File 2: MaxQuant proteinGroups output file containing information for all proteins with one or more unique peptides identified in the study. The tab "proteinGroups" contains information for all of the proteins (n = 231) identified in the experiment, including raw (not log2-transformed) label-free quantification (LFQ) abundance values for each protein. The tab "limma" contains log2-transformed abundances with imputed missing values for all proteins that had two or fewer missing values per sampling point (n = 153) that were used for differential protein abundance analyses (Ritchie *et al.*, 2015).

Supplementary File 3: Output files from limma containing information about differentially abundant proteins (DAPs). The DAPs identified in each comparison are shown in separate tabs. ProteinID refers to the unique, study-specific identifier for each protein.

Description of Raw Data

Raw MS/MS data is available as dataset PXD054543 in the ProteomeXchange MassIVE database: ftp://massive.ucsd.edu/v08/MSV000095508/. Samples were randomized for processing and LC-MS/MS analysis. Sample key for raw data files on ProteomeXchange:

**sample number**	**sample description**
sample-01	seal 6 day 1 pre-ACTH
sample-02	seal 6 day 4 pre-ACTH
sample-03	seal 4 day 1 pre-ACTH
sample-04	seal 4 day 4 8 h post-ACTH
sample-05	seal 6 day 1 24 h post-ACTH
sample-06	seal 4 day 4 4 h post-ACTH
sample-07	seal 6 day 4 4 h post-ACTH
sample-08	seal 4 day 4 pre-ACTH
sample-9	seal 4 day 1 24 h post-ACTH
sample-10	seal 6 day 4 8 h post-ACTH
sample-11	seal 6 day 1 4 h post-ACTH
sample-12	seal 4 day 1 4 h post-ACTH
sample-13	seal 4 day 1 8 h post-ACTH
sample-14	seal 6 day 1 8 h post-ACTH
sample-15	seal 7 day 4 8 h post-ACTH
sample-16	seal 2 day 1 4 h post-ACTH
sample-17	seal 7 day 1 pre-ACTH
sample-18	seal 2 day 4 8 h post-ACTH
sample-19	seal 7 day 1 24 h post-ACTH
sample-20	seal 7 day 4 pre-ACTH
sample-21	seal 7 day 1 8 h post-ACTH
sample-22	seal 2 day 1 8 h post-ACTH
sample-23	seal 7 day 1 4 h post-ACTH
sample-24	seal 7 day 4 4 h post-ACTH
sample-25	seal 2 day 4 pre-ACTH
sample-26	seal 2 day 1 24 h post-ACTH
sample-27	seal 2 day 4 4 h post-ACTH
sample-28	seal 2 day 1 pre-ACTH

References for Supplementary Methods

Deyarmin J, Hekman R, Champagne C, McCormley M, Stephan A, Crocker D, Houser D, Khudyakov J (2020) Blubber proteome response to repeated acth administration in a wild marine mammal. *Comparative Biochemistry and Physiology Part D: Genomics and Proteomics* 33: 100644

Khudyakov JI, Deyarmin JS, Hekman RM, Pujade Busqueta L, Maan R, Mody MJ, Banerjee R, Crocker DE, Champagne CD (2018) A sample preparation workflow for adipose tissue shotgun proteomics and proteogenomics. *Biol Open* 7: bio036731

León IR, Schwämmle V, Jensen ON, Sprenger RR (2013) Quantitative assessment of in-solution digestion efficiency identifies optimal protocols for unbiased protein analysis*. *Molecular & Cellular Proteomics* 12: 2992-3005

Müller T, Winter D (2017) Systematic evaluation of protein reduction and alkylation reveals massive unspecific side effects by iodine-containing reagents*. *Molecular & Cellular Proteomics* 16: 1173-1187

Proc JL, Kuzyk MA, Hardie DB, Yang J, Smith DS, Jackson AM, Parker CE, Borchers CH (2010) A quantitative study of the effects of chaotropic agents, surfactants, and solvents on the digestion efficiency of human plasma proteins by trypsin. *Journal of Proteome Research* 9: 5422-5437

Pujade Busqueta L, Crocker DE, Champagne CD, McCormley MC, Deyarmin JS, Houser DS, Khudyakov JI (2020) A blubber gene expression index for evaluating stress in marine mammals. *Conservation Physiology* 8: coaa082

Ritchie ME, Phipson B, Wu D, Hu Y, Law CW, Shi W, Smyth GK (2015) Limma powers differential expression analyses for rna-sequencing and microarray studies. *Nucleic Acids Research* 43: e47-e47

Scheerlinck E, Dhaenens M, Van Soom A, Peelman L, De Sutter P, Van Steendam K, Deforce D (2015) Minimizing technical variation during sample preparation prior to label-free quantitative mass spectrometry. *Anal Biochem* 490: 14-19

Wojdyla K, Rogowska-Wrzesinska A (2015) Differential alkylation-based redox proteomics – lessons learnt. *Redox Biology* 6: 240-252
